# A cross-sectional study on the quality of life of women with endometriosis in Trinidad and Tobago

**DOI:** 10.3389/fgwh.2024.1359741

**Published:** 2024-08-22

**Authors:** Raveed Khan, Rameez Baksh, Terika J. Wallace, Mikael Aimable, Vineeta Bujhawan, Justin Cumberbatch, Enrie Julian Marbella, Dana Phagoo, Sanathkumar Ramjattan, Akshita Shavili

**Affiliations:** ^1^ Department of Para-Clinical Sciences, Unit of Public Health & Primary Care, Faculty of Medical Sciences, The University of the West Indies St. Augustine, St. Augustine, Trinidad and Tobago; ^2^ Department of Mathematics and Statistics, The University of the West Indies St. Augustine, St. Augustine, Trinidad and Tobago; ^3^ Department of Para Clinical Sciences, Faculty of Medical Sciences, The University of the West Indies St. Augustine, St. Augustine, Trinidad and Tobago

**Keywords:** endometriosis, quality of life, acceptance of illness, pain severity, symptomatology

## Abstract

**Introduction:**

Endometriosis is a chronic gynecological condition that lacks a definitive cure and adversely impacts the quality of life (QoL) of those affected. This study delves into the experiences of individuals with endometriosis in Trinidad and Tobago, focusing on their quality of life, pain severity, and acceptance of illness.

**Methods:**

Surveys were distributed among 160 members of the Trinidad and Tobago Endometriosis Association. The survey instrument utilized was the WHOQOL-BREF, which measures QoL, pain severity, and acceptance of illness [the Acceptance of Illness Scale (AIS)].

**Results:**

The average age of respondents was 38.65 years. Quality of life scores averaged 3.41, with the “environment” aspect scoring highest (12.84) and “social relationships” scoring lowest (11.88). Cronbach's alpha indicated excellent internal consistency for “environment” (*ɑ *= 0.909) and the “AIS” (*ɑ *= 0.882). The independent samples t-test revealed lower mean QoL scores for unemployed participants. Analysis of variance revealed significant differences in mean QoL scores for “health status” and “years since endometriosis diagnosis.” All the QoL domains were positively correlated with each other. There were moderate positive correlations between the physical health and social relationships domains (*ρ *= 0.558). All other domains were strongly correlated with each other (0.6 < *ρ *< 0.8). Pain intensity and acceptance of illness had mean scores of 24.15 and 6.57, respectively. Variations in quality of life were observed for health status and duration since diagnosis, impacting mostly on the domain of physical health. Acceptance of illness emerged as a significant influencer of overall quality of life, assisting individuals in navigating the challenges posed by endometriosis.

**Discussion:**

The findings underscore the importance of understanding determinants, such as pain severity to improve care and support for those with endometriosis. Exploring acceptance of illness is critical in improving the quality of life of these individuals, highlighting the need for tailored interventions that encompass psychological and social support alongside medical treatment. This study demonstrates the pivotal role of acceptance of illness in the overall quality of life of endometriosis patients. Improving the quality of care requires a comprehensive understanding of the factors influencing quality of life, particularly pain severity and the need for a holistic approach to support individuals grappling with endometriosis.

## 1 Introduction

Endometriosis is a common chronic, gynecological, and idiopathic disease identified by the appearance of tissue resembling that of the endometrium outside of the uterus. Endometriosis usually presents with symptoms such as agonizing menstrual cycles, chronic pelvic pain, pain during and/or after sexual intercourse, and depression or anxiety, and is notably a cause of infertility (1, 2). Quality of life (QoL), as defined by the World Health Organization (WHO), is a person's outlook on their position in life according to their cultural background and morals in relation to their goals, expectations, and concerns, which can be used to assess the impact on their life (3). In addition, acceptance of illness (AoI) and severity of pain are predictors of QoL that provide insight into mental and physical discomfort as well as the degree of negative emotions experienced by those with endometriosis (4).

Understanding how endometriosis affects the QoL of patients is important as it can directly influence productivity in the workplace and overall satisfaction with one's life. Increasing knowledge about the impact of endometriosis on QoL can improve public health practices by assisting in updating policies and increasing the quality of care and treatment of endometriosis. In addition, increasing awareness will help reduce the stigmatization of menstrual pain in the workplace and motivate undiagnosed women to seek medical advice and learn how to manage endometriosis efficiently.

Research on this disease is ongoing, with the majority of published articles highlighting its pathophysiology, treatment, and management. However, the impact of this disease on women and how it affects their everyday lifestyle is poorly represented. The WHO estimates that, globally, 10% (190 million) of reproductive age girls and women are affected by endometriosis (5). The UK National Health Service (NHS) has reported that endometriosis is rare in women of African-Caribbean origin and is more common in Asian women than in Caucasian women. This suggests that genes may be involved (6). However, the Jamaican media has reported that more than 100,000 Jamaican women are affected by this condition (7). These statistics represent the estimated prevalence of women diagnosed with endometriosis but are grossly underestimated due to diagnostic and treatment delays, stigmatization, and limited awareness of the disease (8). Prevalence rates in Latin America and the Caribbean are largely unknown, with published data only from Puerto Rico and Chile (9).

A study by Nnoaham et al. focusing on the impact of endometriosis on women and society stated that physical health–related QoL was significantly reduced in diagnosed women compared with undiagnosed women who exhibit similar symptoms. They reported an average diagnostic delay of 6.7 years and that delays were strongly associated with care-seeking experiences in primary care (10). Extensive research has been carried out regarding the positive correlation between QoL and the AoI among chronically ill patients. Conversely, research investigating a relationship among pain severity, QoL, and AoI in women suffering from endometriosis is sparse. Notably, findings from a recent regional study reported that stigma could be one of the mechanisms through which the relationship between incapacitating pain and self-esteem among Latin American and Caribbean women with endometriosis could be partially explained (9). QoL is negatively affected by the presence of endometriosis and thus emphasizes the need for more local studies relating to this topic.

Endometriosis is a chronic disease that impacts multiple aspects of women’s lives mainly because of the difficulty in diagnosing it (11). However, early diagnosis and early treatment may prevent or at least slow down disease progression and have a good impact on QoL and fertility (12). This is particularly important as endometriosis patients may have an increased risk of developing endometriosis-associated ovarian cancer (EAOC), thereby heavily affecting the QoL and reproductive history of these patients (13). Common symptoms include chronic pelvic pain, fatigue, congestive dysmenorrhea, heavy menstrual bleeding, and deep dyspareunia. Studies have demonstrated the considerable negative impact of this condition on women’s QoL, especially in the domains of pain and psychosocial functioning (14).

This study seeks to provide insight into the impact of physician-diagnosed endometriosis on QoL and its predictors of severity of pain and AoI among women over the age of 18 in Trinidad. The aim is to assess how QoL is affected by the severity of pain and AoI among women with physician-diagnosed endometriosis in Trinidad and Tobago.

## 2 Materials and methods

### 2.1 Study setting

This research was conducted virtually from December 2022 to July 2023. Questionnaires were distributed exclusively to women with physician-diagnosed endometriosis who were members of the Trinidad and Tobago Endometriosis Association (TTEA). The TTEA is a registered non-profit organization committed to assisting those affected by endometriosis. Surveys were sent to the TTEA via email, who in turn distributed them to the target group via email and social media platforms, which has proven to be useful for its fast dispersion and cost-effectiveness (15).

### 2.2 Study design

A cross-sectional study was performed. Data collection took place between January and May 2023 and participants were recruited through the TTEA. A survey consisting of four sections was distributed to the sample population to capture relevant information on participants’ demographics, health, and pertinent life factors. Furthermore, a QoL survey, WHOQOL-BREF (3), in conjunction with the Laitinen Pain Scale (16) and the Acceptance of Illness Scale (AIS) developed by Felton et al. (17), were employed to determine these respective factors. The WHOQOL-BREF consists of 26 questions scaled from 0 to 5 on a person's perception of their physical and psychological health over a 2-week period. Scores are graded positively, with higher scores representing a better QoL (13). The Laitinen Pain Scale measures individualistic assessment of pain and comprises four questions, allowing patients to gauge the severity and periodicity of pain, the use of pain medication, and activity impediments (4). Questions are scored from 0, “no problem,” to 4, “the biggest possible problem.” The AIS comprises eight statements regarding the implications of illness. It is rated using a five- point scale, with 1 implying strong agreement (poor ability to cope with the condition) and 5 implying strong disagreement (strong ability to cope with the condition). Furthermore, higher scores correlate with the increasing ability of patients to manage with the constraints of their condition and with a greater psychological wellbeing (4).

### 2.3 Study population

This study targeted women from the TTEA over the age of 18 with physician-diagnosed endometriosis. Questionnaires were distributed to participants who provided informed consent by ticking yes to a consent checkbox that explained the rationale for the survey before it was completed. Information regarding the aim of the study and a guide to answering the surveys were provided to the participants.

### 2.4 Sample size

All members of the TTEA were targeted to aim for a total population sample of 160 participants. Using a prevalence rate of 18% (18), confidence level of 95%, and margin of error of 5%, a minimum sample size of 94 participants was computed after adjusting for a finite population.

### 2.5 Study sample

The study sample included those satisfying the following inclusion criteria:

• Females over the age of 18 years with physician-diagnosed endometriosis and who are members of the TTEA.

The following exclusion criteria were applied:

• Women below the age of 18 years.
• Women who have not been diagnosed with endometriosis by a physician.
• Women who were not members of the TTEA.
• Women who refused to participate in the study.

### 2.6 Ethical considerations

All ethical standards were adhered to, in keeping with the University of the West Indies Campus Ethics Committee (Reference CREC-SA.1872/11/2022), the revised Helsinki Declaration 2000, and approvals for the use of materials by the WHO. Furthermore, participation was optional and anonymous, and the results obtained would only be employed for scientific purposes. Participants were allowed to leave the study at any point in time and were educated about the purpose, benefits, and risks of the study before consenting. Finally, participant confidentiality was maintained throughout due to the collection of sensitive information.

### 2.7 Data collection

Data were collected using a self-administered survey comprising 47 close-ended and open-ended questions. The questionnaire consisted of four sections: section 1, demographic information; section 2, QoL of women diagnosed with endometriosis; section 3, severity of pain; and section 4, AoI. A consent form was provided to participants before the administration of the questionnaire; this provided information on the purpose of research, the participants’ requirements, and potential risks or benefits. The participants were assured of confidentiality and privacy and no personal identifiers were recorded. Participants were informed about the implications of this research and asked to confirm their voluntary participation. Owing to COVID-19 protocols, questionnaires were distributed via Google Forms to our study population of 160 women at the TTEA with physician-diagnosed endometriosis. However, of the intended sample of 94 women at the TTEA, the questionnaire was completed by 54 participants, yielding a response rate of approximately 57.4%.

### 2.8 Data analysis

The data were analyzed using version 26 of the Statistical Package for the Social Sciences (SPSS). Continuous variables were presented with means (M) and standard deviations (SD), and categorical variables were presented with frequencies and percentages. In this study, a Categorical Regression using Alternating Least Squares (CATREG) technique was employed to determine the components impacting the QoL of the study population. Independent sample t-tests and analysis of variance (ANOVA) tests were conducted to determine any significant differences in means for the WHOQOL domains. In addition, Pearson's correlation was used to investigate the strength of the relationship between the WHOQOL domains.

## 3 Results

The mean age of our participants was calculated to be 38.56 (±7.65), ranging from 23 to 55 years old. Further demographical results revealed that most participants received tertiary education (74.1%), were married (51.9%), were gainfully employed (88.9%), perceived their health status as good (63%), did not experience menopause (79.6%), and had been diagnosed with endometriosis for 5 years or less (31.5%). Furthermore, the characteristic of “gainfully employed” had significant differences in mean scores for the physical health, social relationships, and environmental domains. “Health status” had significant differences in mean scores for the physical health, psychological, social relationships, and environmental domains. “Years since endometriosis diagnosis” only had significant differences in the means for the physical health domain (see Table 1).

**Table 1:** Demographic characteristics of the respondents and the *p*-value comparison of means for the WHOQOL domains in women with endometriosis.

Characteristics	*N*	%	Physical health	Psychological	Social relationships	Environment
Gainfully employed			0.029^a^	0.102	0.023^a^	0.043^a^
No	6	11.1				
Yes	48	88.9				
Health status			0.009^b^	0.001^b^	0.018^b^	0.005^b^
Very poor	1	1.9				
Poor	7	13				
Neither good nor poor	12	22.2				
Good	34	63				
Years since endometriosis diagnosis			0.041^b^	0.75	0.437	0.469
5 years or less	17	31.5				
6–10 years	11	20.4				
11–15 years	15	27.8				
More than 15 years	11	20.4				

^a^ Significant *p*-value (less than 5%) for the independent sample *t*-test. Mean for >15 significantly different to other groups.

^b^ Significant *p*-value (less than 5%) for ANOVA.

The internal consistency of the different domains was assessed using Cronbach's alpha. The results showed acceptable internal consistency for the physical health (*α* = 0.710), psychological (*α* = 0.725), social relationships (*α* = 0.740), and pain intensity (*α* = 0.797) domains, and excellent internal consistency for the environment domain (*α* = 0.909). In addition, the AIS (*α* = 0.882) demonstrated good internal consistency (see Table 2).

**Table 2:** Cronbach’s alpha values calculated for each specific characteristic tested in the questionnaire.

Domain	Cronbach's alpha
Physical health	0.71
Psychological	0.73
Social relationships	0.74
Environment	0.91
AIS	0.88
Pain intensity	0.80

Among the participants, the general QoL score was 3.41 ± 0.84. The general health score was 3.46 ± 0.79, the physical health score was 12.57 ± 2.29, the psychological domain score was 12.72 ± 2.72, the social relationships score was 11.88 ± 3.96, and the environment domain score was 12.84 ± 3.46 (see Table 3).

**Table 3:** WHOQOL scores for the general health, physical health, psychological, social relationship, and environment domains in women with endometriosis in TTEA.

Domains	Mean	Median	SD
General quality of life	3.41	3.50	0.84
General health	3.46	4.00	0.79
Physical health	12.29	12.57	2.29
Psychological	12.72	12.33	2.72
Social relationship	11.88	12.00	3.96
Environment	12.84	13.25	3.46

SD, standard deviation.

Notably, the mean AoI score was 24.15 ± 7.51 and the mean pain intensity score was 6.57 ± 2.98 (see Table 4). All the QoL domains were positively correlated with each other. There were moderate positive correlations between the physical health and social relationships domains (*ρ *= 0.558). All other domains were strongly correlated with each other (0.6 < *ρ *< 0.8) (see Table 5).

**Table 4:** Pain intensity and acceptance of illness mean values of women with endometriosis in TTEA.

Statement	Score		
	Mean	Median	SD
Mean acceptance of illness score	24.15	24.00	7.51
Mean pain intensity score	6.57	6.00	2.98

**Table 5:** Pearson's correlation for WHOQOL domains.

		Physical health	Psychological	Social relationships	Environmental
Physical health	Correlation coefficient	1	0.722^a^	0.578^a^	0.749^a^
	Significance value		<0.001	<0.001	<0.001
	*N*	54	54	54	54
Psychological	Correlation coefficient	0.722^a^	1	0.687^a^	0.683^a^
	Significance value	<0.001		<0.001	<0.001
	*N*	54	54	54	054
Social relationship	Correlation coefficient	0.578^a^	0.687	1	0.683^a^
	Significance value	<0.001	<0.001		<0.001
	*N*	54	54	54	54
Environmental	Correlation coefficient	0.749^a^	0.683^a^	0.683^a^	1
	Significance value	<0.001	<0.001	<0.001	
	*N*	54	54	54	54

^a^ Significant correlation at <0.01.

**Figure 1:**
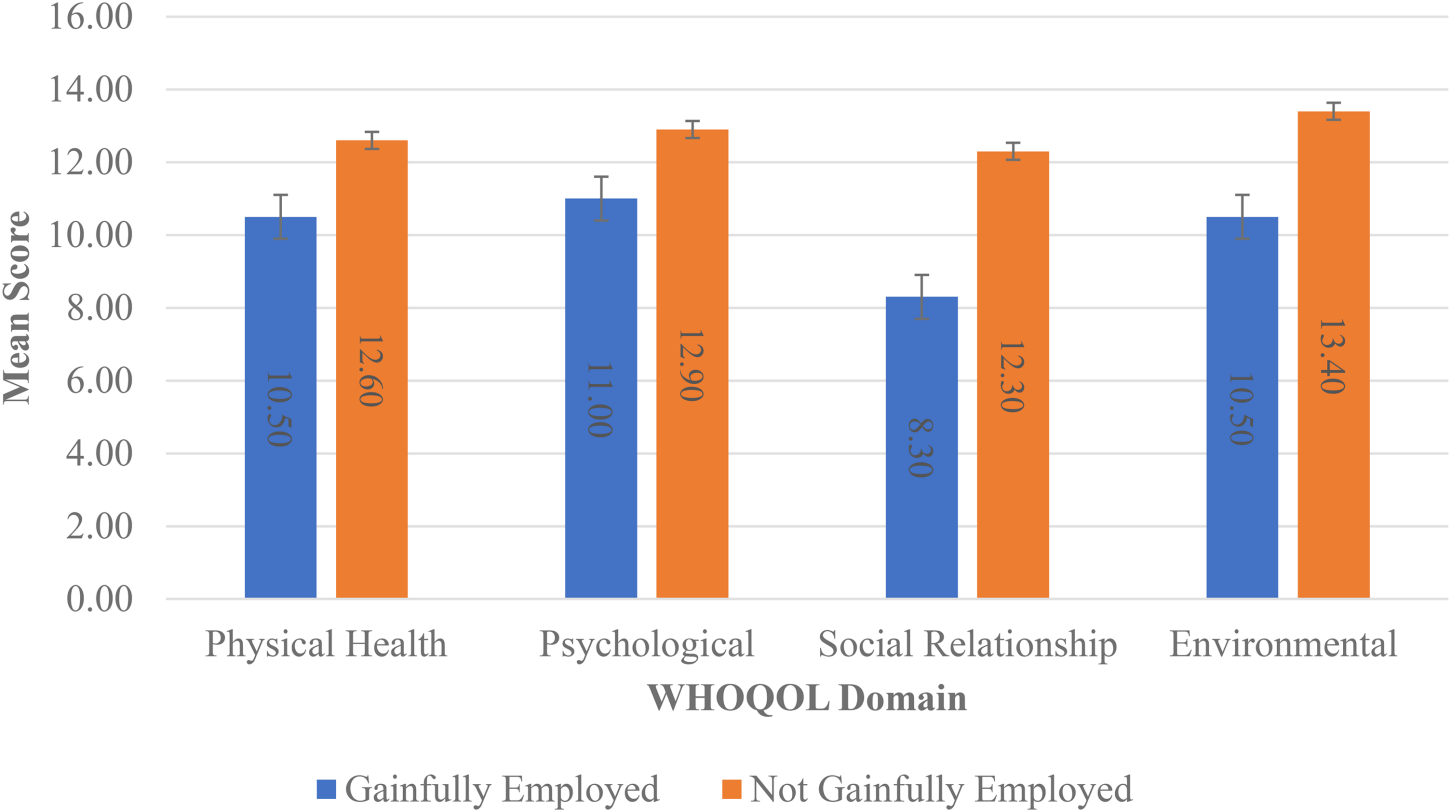
Mean scores for WHOQOL domains by employment status.

**Figure 2:**
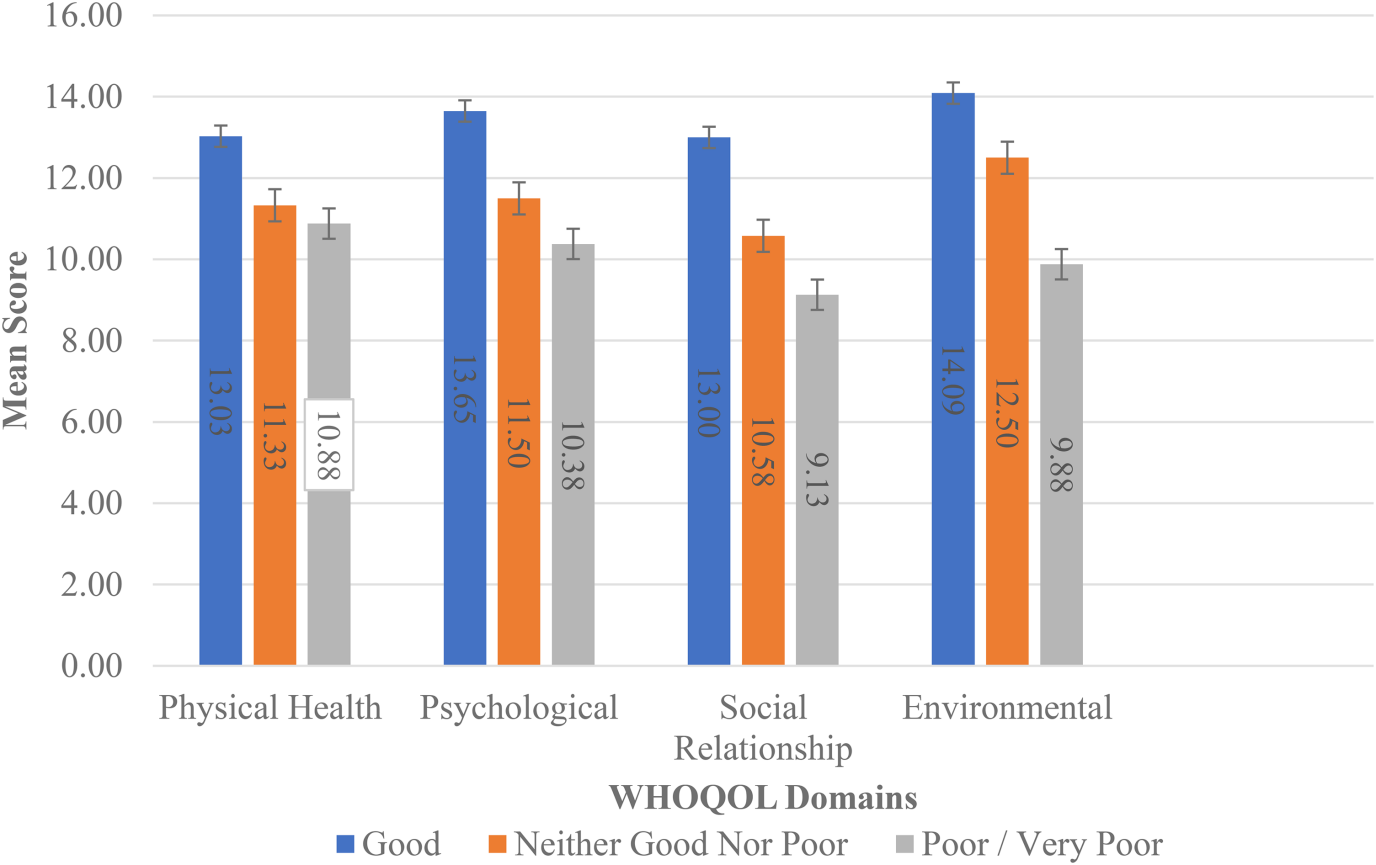
Mean scores for WHOQOL domains by health status.

There were significant differences (*p *< 0.05) in the means for patients by employment status for the physical health, social relationships, and environmental domains of QoL. The independent samples t-test confirmed that patients not gainfully employed had lower means than those gainfully employed (see Figure 1). ANOVA indicated significant differences in the means (*p* < 0.05) for all domains by health status, and the *post-hoc* least significant difference (LSD) test indicated that the means for the health status characteristic were significantly different from each other (see Figure 2).

**Figure 3:**
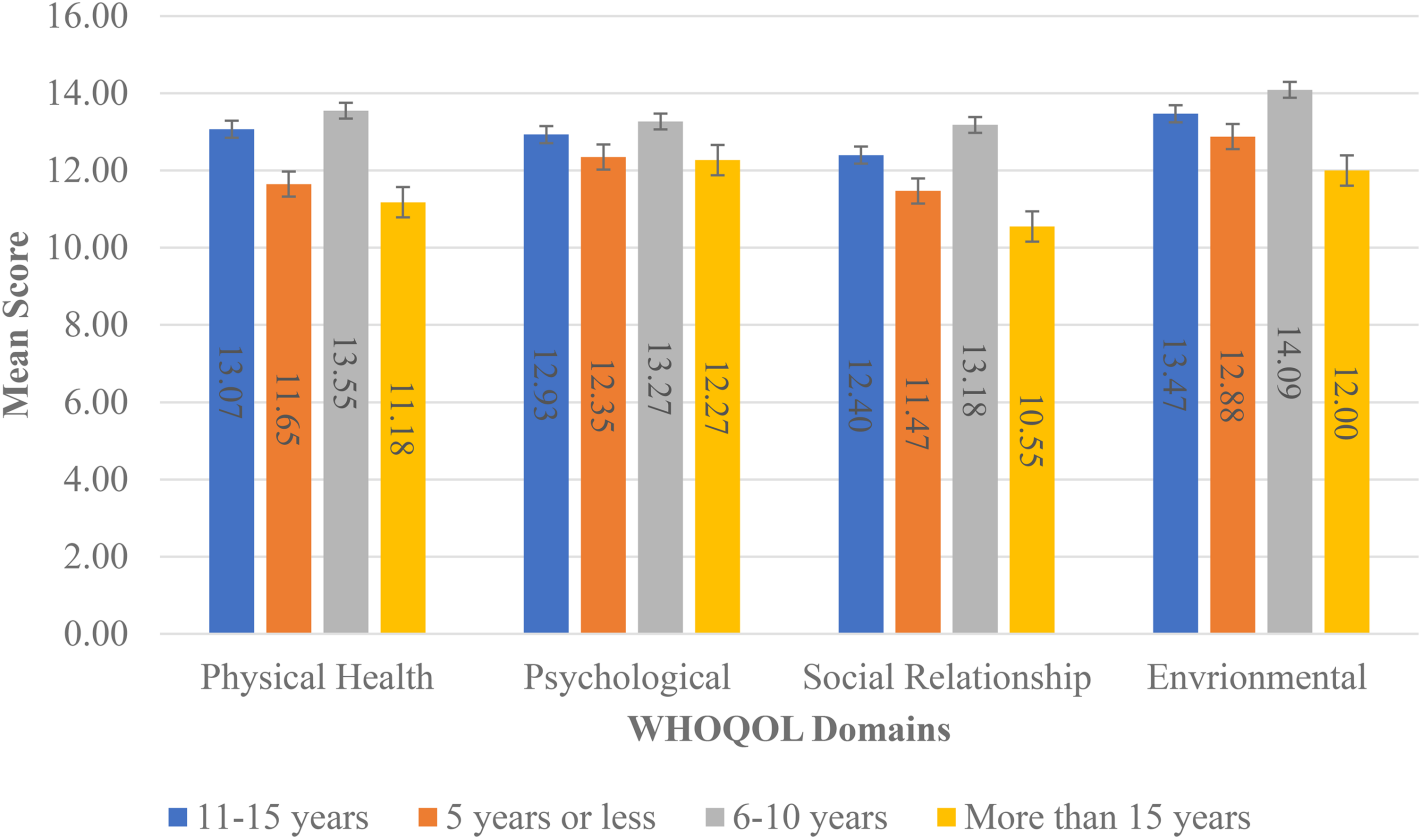
Means by years since endometriosis diagnosis by WHOQOL domains.

ANOVA indicated a statistically significant difference in the means (*p* = 0.041) for years since endometriosis diagnosis and the physical health domain. The *post-hoc* LSD test indicated that the means for the category >15 years was significantly different from the other categories. In the physical health domain, patients diagnosed for more than 15 years had the lowest WHOQOL score (lower scores indicate a lower QoL) compared with the other categories (see Figure 3).

## 4 Discussion

This study aimed to assess how QoL is affected by the severity of pain and AoI among women with physician-diagnosed endometriosis in Trinidad and Tobago. The main objective was to recognize which features of QoL are mainly affected by endometriosis and its correlation with severity of pain and AoI.

The main findings of this study were an average QoL score of 3.41, with the “environment” aspect scoring highest (12.84) and “social relationships” scoring lowest (11.88). The independent samples t-test revealed lower mean QoL scores for unemployed participants. ANOVA revealed significant differences in mean QoL scores for “health status” and “years since endometriosis diagnosis.” All the QoL domains were positively correlated with each other. There were moderate positive correlations between the physical health and social relationships domains (*ρ *= 0.558). All other domains were strongly correlated with each other (0.6 < *ρ *< 0.8). Pain intensity and AoI had mean scores of 24.15 and 6.57, respectively. Variations in QoL were observed for health status and duration since diagnosis, impacting mostly on the domain of physical health.

Through analysis of the WHOQOL domains by health status it is evident that a better health status yields a better mean WHOQOL score for each respective domain, reiterating that health status is influenced by a combination of all the domains of QoL. Bień et al. reveal that the lowest QoL scores in women suffering from endometriosis were for the physical domain; the main problems being pain, the regular intake of medication required to function normally, dissatisfaction with their performance in daily life, and productivity (4). Contrastingly, our study recorded the lowest QoL scores in the social relationships domain (11.88 ± 3.96). This could be on account of painful pelvic cramps and/or intercourse, which can affect interpersonal relationships, sex life, and variations in support systems available to patients, many of which may be less empathetic and sympathetic to women with endometriosis. An Australian study conducted by Moradi et al. reported that dyspareunia was a common symptom that had a major impact on sexual/marital relationships, which led to broken relationships for some women (19).

Interestingly, our findings in the physical health domain revealed that patients diagnosed for more than 15 years had the lowest WHOQOL score. This is due to these participants having to withstand longer periods of endometriosis symptomology inclusive of pain and intense prolonged treatment regimens. In addition, we recognized that patients who were not gainfully employed reported lower mean scores for the physical health, social relationships, and environmental domains of QoL. This shows that unemployed endometriosis patients tend to have poorer physical health, which influences socialization and interpersonal interactions with their community. This study also investigated the AIS, which provides greater insight into the patient’s adjustment to their health condition and investigates how efficiently they perform their everyday activities while enduring endometriosis-associated symptoms. Patients who have adapted better to this condition tend to have a lower level of discomfort and as such a higher AIS score. These patients have adjusted better to the emotional and physical discomforts and as such may have lower levels of stress, better mental health, and higher self-esteem. The mean AIS score obtained in our study was 24.15, which denotes a moderate level of AoI. This result is in keeping with that found in the study conducted by Bień et al., in which the study participants had an AIS score of 24.64 (4). These scores suggest that participants have partly adjusted to the negative consequences of endometriosis.

In our study, participants appeared to have endured a moderate pain intensity, scoring an average of 6.57 out of 16, yet had a high AoI on their ability to function regardless of the complications associated with their disease. Our research reflects that the QoL domain that was statistically significant was the psychological aspect. AoI, marital status, and health were all confirmed predictors of how the psychological domain could alter QoL. Thus, a negative AIS score (*β *= −0.512) would indicate that the study population had a lower negative psychological impact and a more positive health score (*β *= 0.401), indicating that the study population was less psychologically impacted and therefore less likely to develop depression and anxiety and could enjoy life to an extent. Notwithstanding our findings, it should be noted that an Austrian study by Friedl et al. reported that 15.1% of women with endometriosis in the study group had been diagnosed with depression. In addition, endometriosis along with pelvic pain increases the occurrence of symptoms associated with depression more than endometriosis without pain (20). Arena et al. reported that women's anxiety levels were higher when seeking information online and during the diagnostic phase of the disease (21). This underscores the need for physicians to be more empathic in their consultations as this would assist in decreasing anxiety levels and reduce the burden of the disease from its outset.

The significant correlation between QoL and psychological aspects can serve as a basis for informing the choice of therapy. Indeed, pain symptoms may result from a combination of nociceptive (pain caused by damage to body tissue), neuropathic (pain caused by a lesion or disease of the somatosensory nervous system), and nociplastic pain (pain arising from altered nociception without evidence of tissue or somatosensory damage). Approximately 25% of individuals with endometriosis do not respond to hormonal treatment and surgery, underscoring a need for the implementation of a suitable screening questionnaire tool that could assist clinicians in choosing appropriate therapies and informing the development of personalized management strategies (22). Central sensitization, which is a particular type of nociplastic pain, has a high prevalence in patients with endometriosis, especially in those with moderate to severe chronic pelvic pain (23), and can potentially result in the diagnosis of other conditions or delayed diagnosis, thereby worsening QoL.

### 4.1 Limitations

Limitations include a low response rate and self-report bias. Despite persistent communications sent to the target population, there was a relatively low number of responses. This contributes to non-response bias as those who did not respond may have different characteristics to those who responded. The results of the study may not be representative of the study population and may therefore lead to potential generalizations that are not reflective of the general population. Second, a potential self-report bias may be present among participants due to individual interpretations of questions, an inability to recall past experiences accurately, or a social desirability bias that could lead to an underestimation of stigmatizing attitudes or an overestimation of socially acceptable perspectives (24). Additional limitations of online dissemination include technical and connectivity issues and the potentially low technological literacy of the respondents.

## 5 Conclusion

QoL and its predictors of AoI and severity of pain are important factors to consider in endometriosis, as they provide insight into the effectiveness of the available diagnostic procedures, treatment plans, and general patient adjustments in living with this disease. Our study revealed that each domain of the QoL is affected differently in relation to severity of pain and AoI, and therefore the diagnosis, treatment, and management of the disease should be individualized yet multidisciplinary in their approach to achieve optimal patient care. Patient's QoL scores were similar to those of their perceived general health. The social relationships domain was the most affected and the environmental domain was the least affected. In particular, factors affecting the QoL of women diagnosed with endometriosis can be attributed to the joint impact of a moderate AoI and very high pain severity, which exacerbates factors such as painful pelvic cramps and/or intercourse, which can strain interpersonal relationships, sex life, and support systems. This study increases the understanding and appreciation for the women in our country who are plagued by this disease and will aid in reducing diagnostic delays and improving access to better quality care. It is evident that the goal should be to improve the overall quality of these women's lives and as such management should extend beyond symptomatic management and adopt a holistic approach, incorporating the social, emotional, and mental implications of this disease. It is believed that such an approach will undoubtedly improve the overall QoL of women suffering from endometriosis in Trinidad and Tobago.

### 5.1 Recommendations

• Public health forums should be hosted to raise awareness of endometriosis and its symptomatology, diagnosis, and treatment.
• Public health education on endometriosis should include its impact on the QoL of women suffering from the disease, including all aspects of life that are affected: mental, physical, emotional, and social.
• Treatment programs for endometriosis should be multidisciplinary in their approach and provide individualized and holistic care.

## Data Availability

The raw data supporting the conclusions of this article will be made available by the authors, without undue reservation.
