# Supplementary material for: A cross-sectional study on the quality of life of women with endometriosis in Trinidad and Tobago
Source: Front Glob Womens Health. 2024 Aug 22;5:1359741. doi: 10.3389/fgwh.2024.1359741 (PMC11374614; doi:10.3389/fgwh.2024.1359741)
Supplement: Supplementary file 1 [file Datasheet1.docx]

**UWI Ethics Approval**


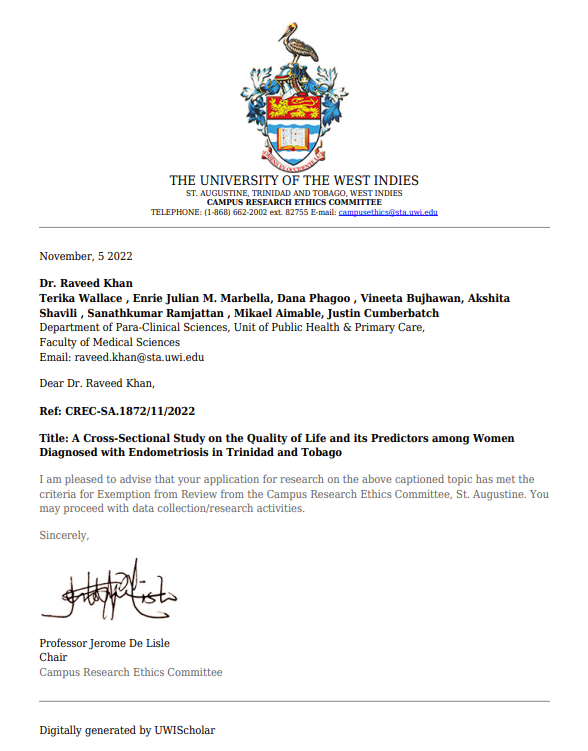


***Informed Consent Form***

***
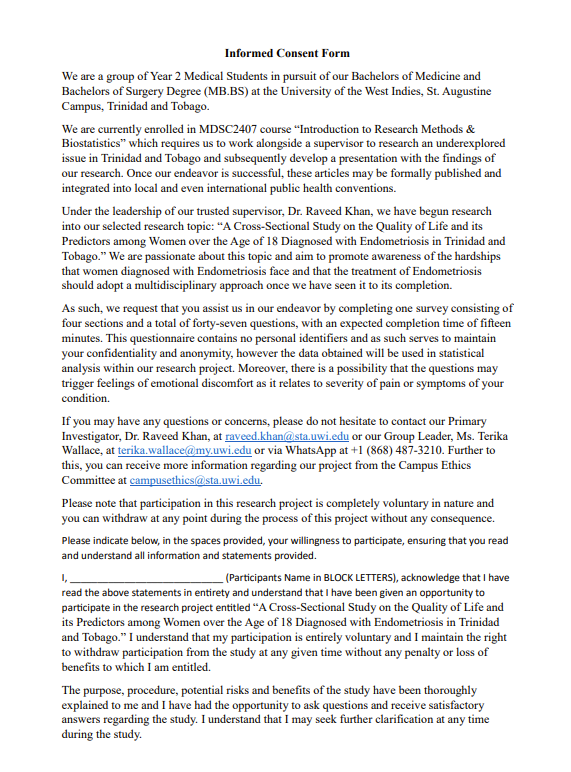
***

***
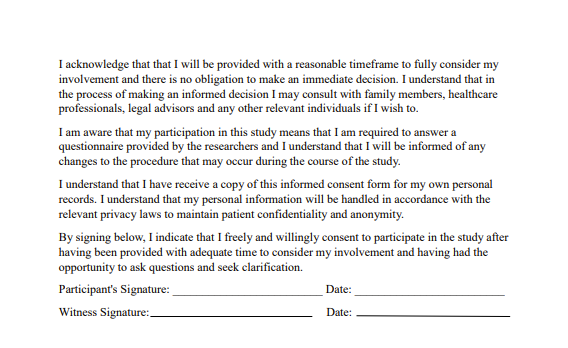
***

**Questionnaire**


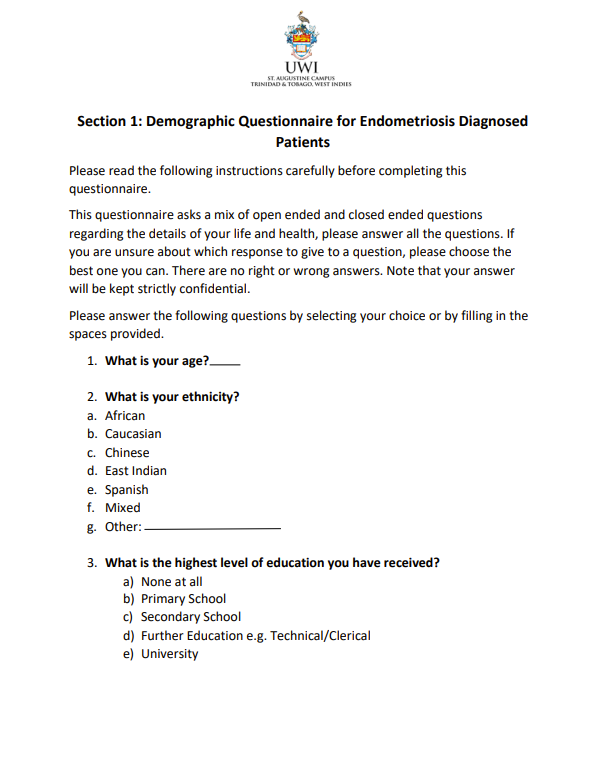


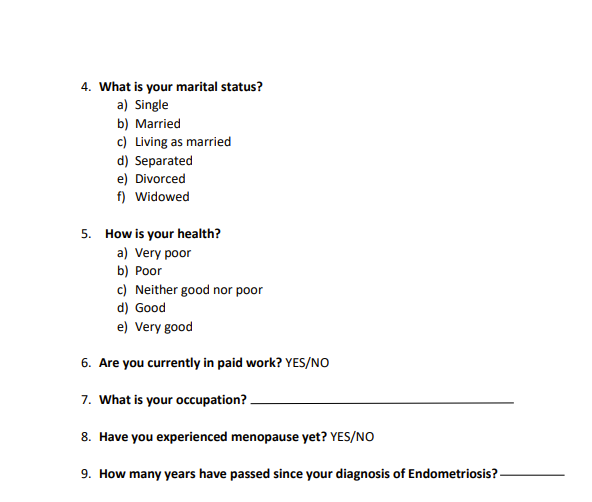


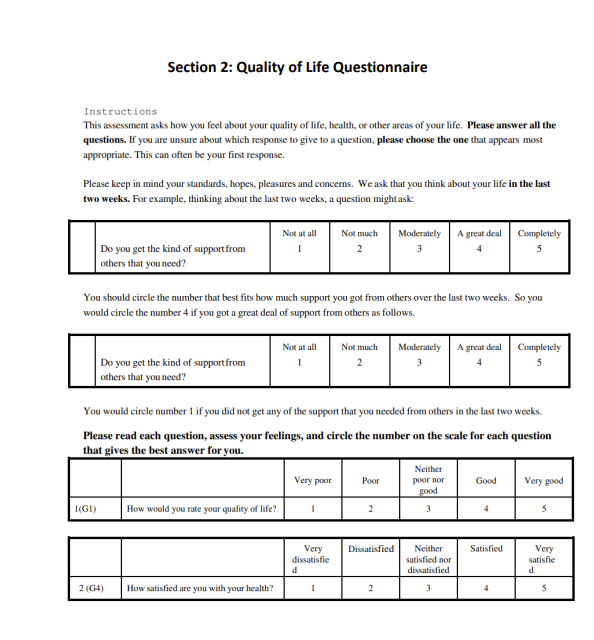


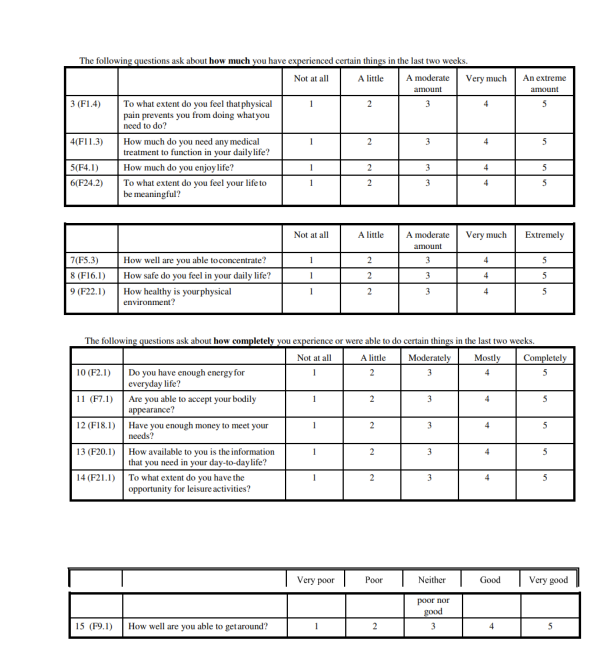


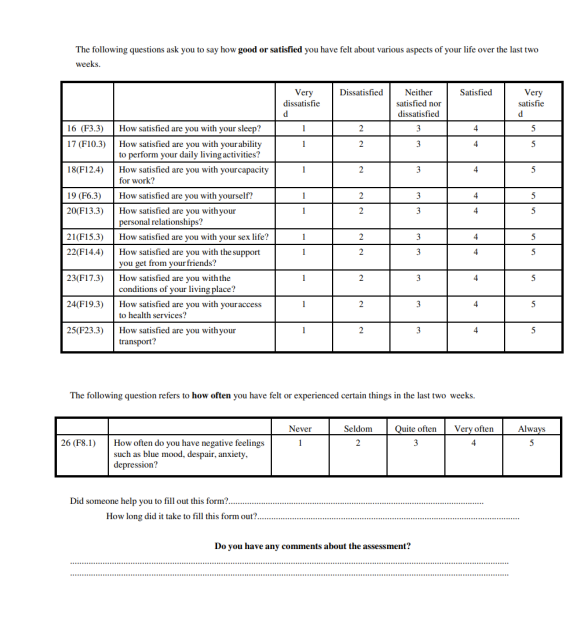


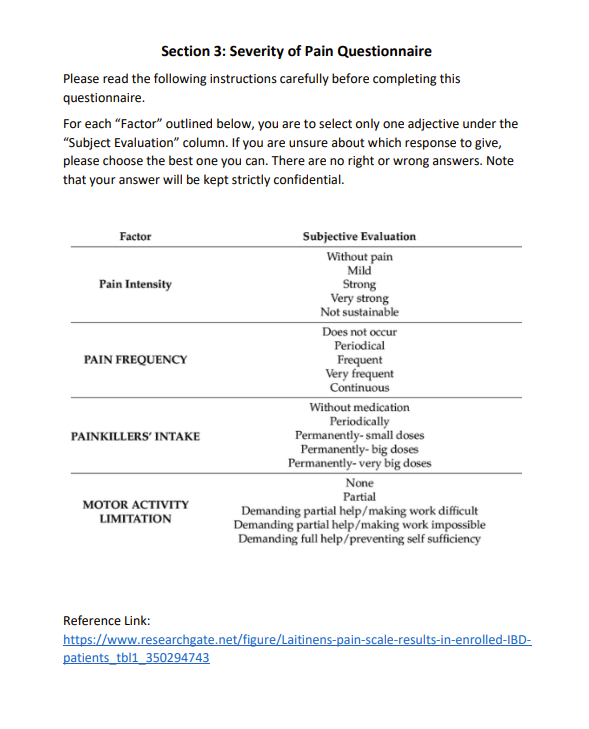


Reference Link:

<https://www.researchgate.net/figure/Laitinens-pain-scale-results-in-enrolled-IBD-patients_tbl1_350294743>


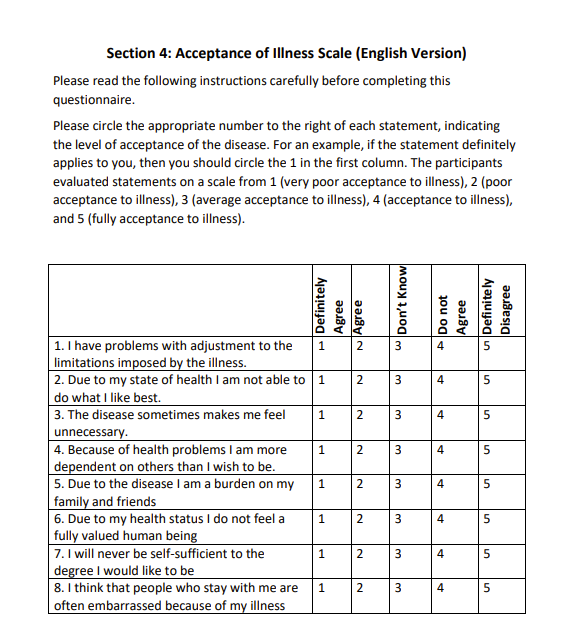


Reference Link:

https://pubmed.ncbi.nlm.nih.gov/34587199/
